# The Role of the Metabolic Parameters of ^18^F-FDG PET/CT in Patients With Locally Advanced Cervical Cancer

**DOI:** 10.3389/fonc.2021.698744

**Published:** 2021-08-19

**Authors:** Dunhuang Wang, Xiaoliang Liu, Weiping Wang, Li Huo, Qingqing Pan, Xue Ren, Fuquan Zhang, Ke Hu

**Affiliations:** ^1^Department of Radiation Oncology, The First Affiliated Hospital of Xiamen University, Teaching Hospital of Fujian Medical University, Xiamen, China; ^2^Department of Radiation Oncology, Peking Union Medical College Hospital, Chinese Academy of Medical Sciences & Peking Union Medical College, Beijing, China; ^3^Department of Nuclear Medicine, Peking Union Medical College Hospital, Chinese Academy of Medical Sciences & Peking Union Medical College, Beijing, China; ^4^Department of Radiology, Xiamen Humanity Hospital, Xiamen, China

**Keywords:** cervical cancer, ^18^F-FDG PET/CT, metabolic tumor volume, total lesion glycolysis, radiotherapy

## Abstract

**Purpose:**

To evaluate the role of the pre-treatment cervical and lymph node (LN) metabolic parameters of ^18^F-fluorodeoxyglucose positron emission tomography-computed tomography (^18^F-FDG PET/CT) for locally advanced cervical cancer (LACC) patients receiving concurrent chemoradiotherapy or radiotherapy.

**Methods:**

we reviewed 125 consecutive patients with LACC who underwent pre-treatment ^18^F-FDG PET/CT examination and concurrent chemoradiotherapy or radiotherapy from February 2010 to December 2015 at our institute. The mean standardized uptake value (SUVmean), maximum standardized uptake value (SUVmax), metabolic tumor volume (MTV), and total lesion glycolysis (TLG) of cervical lesion and lymph node (LN) were recorded. Receiver operator characteristic curve, C-index, Kaplan-Meier method, and Cox proportional hazards models were performed.

**Results:**

The median follow-up was 62 months (range, 4-114 months). For 125 included patients with cervical cancer, the 5-year overall survival (OS), disease-free survival (DFS), local control (LC) and distant metastasis-free survival (DMFS) rates were 83.6%, 75.1%, 92.3% and 79.9%, respectively. Cervical MTV (c-index 0.59-0.61) and cervical TLG (c-index 0.60-0.62) values calculated with a threshold of 40% SUVmax presented stronger prediction capability than cervical SUVmean (c-index 0.51-0.58) and cervical SUVmax (c-index 0.53-0.57) for OS, DFS, LC, and DMFS. In univariate analysis, cervical TLG ≥ 113.4 had worse DFS and DMFS. Cervical MTV ≥ 18.3 cm^3^ had worse OS and DMFS. In multivariate analysis, cervical TLG ≥ 113.4 implied worse OS, DFS, and DMFS. In either univariate or multivariate analyses, cervical SUVmean and cervical SUVmax had no statistically significant correlation with OS, DFS, LC and DMFS. For 55 cervical cancer patients with positive LN, LN SUVmax presented strongest prediction capability for OS (c-index = 0.79), DFS (c-index = 0.72), LC (c-index = 0.62), and DMFS (c-index = 0.79). In multivariate analysis, LN SUVmax remained significant biomarker linked to OS, DFS, and DMFS.

**Conclusion:**

Pre-treatment cervical and LN metabolic parameters were associated with survival outcomes in patients with LACC. In our study, we found that pre-treatment cervical TLG and LN SUVmax may be important prognostic biomarkers for OS, DFS, and DMFS. However, further prospective studies with a large number of patients are required to evaluate the value of the metabolic parameters in survival outcomes prediction.

## Introduction

Cervical cancer is a global health problem and the leading cause of cancer death for women in developing countries (1). Cervical cancer ranks eighth in incidence and mortality in China (2). Almost half of the patients present with locally advanced disease at the time of diagnosis. Currently, the primary therapeutic method for patients with locally advanced cervical cancer (LACC) is concurrent chemoradiotherapy. In approximately 80% of patients with disease recurrence, disease failure of cervical cancer occurs within 2 years after initial treatment. Some prognostic factors have been associated with clinical outcomes, including age, stage, tumor pathology, primary tumor size, lymph node status, squamous cell carcinoma antigen, and human papillomavirus (3–6).

^18^F-fluorodeoxyglucose positron emission tomography-computed tomography (^18^F-FDG PET/CT) has become an essential imaging tool in oncology in addition to conventional radiologic methods such as computed tomography (CT) and magnetic resonance imaging (MRI) (7). It is widely used in the diagnosis, clinical staging, response evaluation, curative effect observation, failure mode and prognostic analysis of cervical cancer and other tumors (8–12). In recent years, the association between the metabolic parameters of pre- and post-treatment ^18^F-FDG PET/CT and treatment failure or survival in cervical cancer has become a research hotspot. Metabolic parameters, such as the mean standardized uptake value (SUVmean), maximum standardized uptake value (SUVmax), metabolic tumor volume (MTV), and total lesion glycolysis (TLG), are the focus of attention (13). On the one hand, some studies have reported the correlations between metabolic parameters and the clinical outcomes of cervical cancer. Patients with cervical cancer with a high SUVmax primary lesion show a worse prognosis (14, 15). The baseline SUVmean can effectively predict the histopathological partial response of the primary tumor in LACC patients treated with chemoradiotherapy followed by surgery, suggesting the potential role of ^18^F-FDG PET/CT in personalized treatment (16). Pre-treatment MTV and TLG are predictors of response to therapy and are correlated with overall survival in cervical cancer patients treated with chemoradiotherapy (17, 18). On the other hand, there are some dissenting views. The role of SUVmax and SUVmean as prognostic factors for cervical cancer is still controversial (19). Whether MTV and TLG are important prognostic indicators of cervical cancer remains to be further studied (20).

In this study, we reviewed cervical cancer patients with pre-treatment ^18^F-FDG PET/CT and analyzed the associations between metabolic parameters and treatment failure or survival.

## Methods

### Patients

We reviewed patients with LACC who received a pre-treatment ^18^F-FDG PET/CT scan and were treated with concurrent chemoradiotherapy or radiotherapy between February 2010 and December 2015 at our institute. The inclusion criteria were as follows: (1) pathologically proven cervical cancer; (2) 2009 FIGO stage IB2, IIA2 and IIB-IVA; (3) underwent ^18^F-FDG PET/CT scan before primary treatment; (4) no evidence of distant metastases; and (5) treated with concurrent chemoradiotherapy or radiotherapy. The exclusion criteria were as follows: (1) underwent conization of the cervix; (2) previous or concurrent diagnosis of secondary primary tumor; (3) Karnofsky performance score <70; and (4) diagnosis of diabetes mellitus.

Pre-treatment evaluations included history, physical, and gynecological examinations, complete blood count, liver function test, renal function studies, chest and abdomen CT or whole-body PET/CT, and pelvic MRI.

### PET/CT Technique and Image Analysis

The imaging agent ^18^F-FDG, which has both a radiochemical purity and chemical purity greater than 98% and negative 24 h bacterial culture and bacterial endotoxin test by the gel method, was synthesized by the PET center of Peking Union Medical College Hospital. All patients fasted for at least 4 hours and rested for 90 minutes before an intravenous administration of 3.7-7.4 (0.1-0.2 mCi) MBq/kg body weight ^18^F-FDG. The blood glucose level was less than 8 mmol/l before the administration of the radiotracer. The PET images were acquired in 3-dimensional mode with a Siemens Biograph 64 PET/CT system from the skull base to the symphysis pubis 1 hour after injection.

The acquired data were reconstructed using the ordered-subset expectation maximization method (two iterations, eight subsets, Gaussian filter, image matrix size 168 × 168). The attenuation-corrected volumetric images were collected in axial, coronal, and sagittal views, and they were independently performed by 2 senior PET physicians. The readers came to a consensus over controversial viewpoints. The SUVmax of the primary lesion of the cervix or positive lymph node (LN) was measured. A contouring around the primary cervical lesions or positive LN inside the boundaries was automatically determined, and the region of interest (ROI) with 40% SUVmax of the primary lesion of the cervix or positive LN within the contouring margin was delineated to define the cervical or LN MTV (20). The TLG of the primary lesion of the cervix or positive LN was calculated by multiplying the cervical or LN MTV by its SUVmean. The LN MTV and LN TLG analyzed in this study were calculated from the most FDG-avid lymph node (21).

### Treatment

All patients were treated with external beam radiation therapy (EBRT) and high-dose-rate brachytherapy. EBRT was delivered with intensity-modulated radiation therapy (IMRT), volumetric-modulated arc therapy (VMAT), or helical tomotherapy (HT). A total of 50.4 Gy external radiation (1.8 Gy per fraction daily) was delivered to the elective regional lymphatics, and 59.36-61.6 Gy (2.12-2.2 Gy per fraction daily) was prescribed for the positive lymph nodes with simultaneous integrated boost (SIB) targets. For patients with para-aortic nodal involvement, the superior border extended to the level of renal vessels or to the upper margin of T12. High-dose-rate brachytherapy was delivered with an Ir-192 source, with 24-36 Gy (biologically effective dose 38.4-57.6 Gy) in four to six fractions to point A. The first-line recommendation of concurrent chemotherapy was weekly cisplatin (40 mg/m^2^). A small number of patients received radical radiotherapy alone.

### Follow-Up

Patients underwent follow-up examinations every 3 months in the first 2 years, every 6 months from 3 to 5 years, and once per year thereafter. Disease failure was confirmed by pathology or evidence of disease recurrence based on a series of imaging data. Overall survival (OS) was defined as the time from the start of treatment to death from any cause or the last follow-up. Disease-free survival (DFS) was defined as the time from the end of treatment to recurrence or the last follow-up. Local control (LC) was defined as the time from the end of treatment to local failure or the last follow-up. Distant metastasis-free survival (DMFS) was defined as the time from the end of treatment to distant metastasis or the last follow-up.

### Statistical Analysis

R and SPSS software (version 26.0; SPSS Inc., Chicago, Illinois, USA) were used for statistical analyses. ROC curve analysis was performed to determine the cut-off values of SUVmax, SUVmean, MTV and TLG of the primary lesion of the cervix or positive lymph node that indicated the optimal trade-off by maximizing the sum of sensitivity and specificity for survival outcomes. The c-index values of SUVmean, SUVmax, MTV, and TLG of the primary lesion of the cervix or lymph node were calculated to present the prediction capability of the metabolic parameters for survival outcomes. The Kaplan–Meier method was used to estimate OS, DFS, LC, and DMFS. Univariate and multivariate analyses of the patient characteristics were performed using the log-rank test and Cox proportional hazards model. To avoid many of the previously significant relationships falling out of the 0.05 significance level, we decided to include variables with p < 0.1 values in the multivariate analysis, and p < 0.05 values were statistically significant.

## Results

A summary of the detailed characteristics of all patients is shown in **Table 1**. In accordance with the inclusion and exclusion criteria of the study, 125 of the 1560 patients were finally included in this study. A total of 112 patients (89.6%) presented with stage IIB or above cervical cancer. A total of 114 patients (91.2%) had squamous cell carcinoma, 9 patients (7.2%) had adenocarcinoma, 1 patient had clear cell carcinoma, and the remaining patient had Mullerian carcinosarcoma. Fifty-two patients (41.6%) had a tumor size greater than 4 cm by gynecological examination. Forty-three patients (34.4%) had positive pelvic metastatic lymph nodes (MLNs) and 2 patients (1.6%) had positive para-aortic MLNs confirmed by ^18^F-FDG PET/CT; 10 patients (8%) with positive para-aortic MLNs had concomitant pelvic lymph nodes metastasis.

**Table 1:** Characteristics for study patients.

Characteristic	Number of patients	Percent of patients
**Median age, y**	50 (range, 30-81)	
**2009 FIGO stage**		
IB	10	8%
IIA	3	2.4%
IIB	84	67.2%
IIIA	3	2.4%
IIIB	24	19.2%
IVA	1	0.8%
**Histology**		
Squamous cell carcinoma	114	91.2%
Adenocarcinoma	9	7.2%
Others	2	1.6%
**Primary tumor size**		
≤4 cm	73	58.4%
>4 cm	52	41.6%
**Pelvic MLNs**		
Yes	53	42.4%
No	72	57.6%
**Para-aortic MLNs**		
Yes	12	9.6%
No	113	90.4%
**Treatment duration**		
≤56 days	96	76.8%
>56 days	29	23.2%
**Total point A EQD_2Gy_**		
<85 Gy	14	11.2%
≥85 Gy	111	88.8%
**Concurrent chemoradiotherapy**		
Yes	102	81.6%
No	23	18.4%
**Cervical SUVmean**		
<7.9	74	59.2%
≥7.9	51	40.8%
**Cervical SUVmax**		
<12.8	68	54.4%
≥12.8	57	45.6%
**Cervical MTV**		
<18.3 cm^3^	59	47.2%
≥18.3 cm^3^	66	52.8%
**Cervical TLG**		
<113.4	55	44%
≥113.4	70	56%
**Lymph node SUVmean**		
<2.2	29	52.7%
≥2.2	26	47.3%
**Lymph node SUVmax**		
<6.7	43	78.2%
≥6.7	12	21.8%
**Lymph node MTV**		
<9.8 cm^3^	47	85.5%
≥9.8 cm^3^	8	14.5%
**Lymph node TLG**		
<6.8	30	54.5%
≥6.8	25	45.5%

FIGO, International Federation of Gynecology and Obstetrics; MLN, metastatic lymph node; EQD_2Gy_, equivalent dose at 2 Gy; SUV, standardized uptake value; MTV, metabolic tumor volume; TLG, total lesion glycolysis.

All 125 patients completed concurrent chemoradiotherapy or radiotherapy with a median time of 51 days (range, 42-98 days). Twelve patients (9.6%) received neoadjuvant chemotherapy followed by chemoradiotherapy or radiotherapy alone, 102 patients (81.6%) received concurrent chemoradiotherapy as the primary therapy, and the remaining 11 patients (8.8%) received radiotherapy alone. Ninety-seven patients (77.6%) completed more than or equal to four cycles of chemotherapy. A total of 111 patients (88.8%) underwent a total point A equivalent dose at 2 Gy (EQD_2Gy_) greater than or equal to 85 Gy.

**Figure 1:**
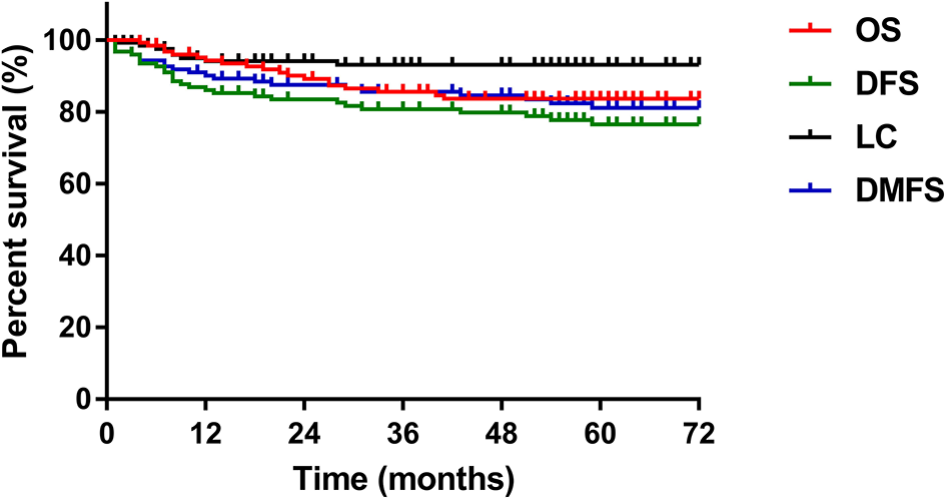
The 5-year OS, DFS, LC, and DMFS curves of the study patients. OS, overall survival; DFS, disease-free survival; LC, local control; DMFS, distant metastasis-free survival.

The median follow-up period for all patients was 62 months (range, 4-114 months). Of the 125 patients, 24% (n=30) experienced disease failure, including 6 patients with pelvic failure, 21 patients with distant metastases, and 3 patients with concurrent local and distant progression. The total local recurrence and distant failure rates were 7.2% and 19.2%, respectively. The cervix uterus was the most common site of pelvic recurrence, and the lung was the most common site of distant metastases. The 5-year overall survival, disease-free survival, local control and distant metastasis-free survival rates were 83.6%, 75.1%, 92.3% and 79.9%, respectively (**Figure 1**).

**Figure 2:**
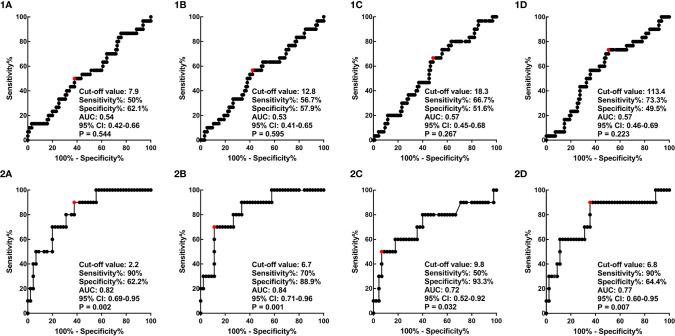
ROC curves of cervical SUVmean **(1A)**, cervical SUVmax **(1B)**, cervical MTV **(1C)**, and cervical TLG **(1D)**, and ROC curves of LN SUVmean **(2A)**, LN SUVmax **(2B)**, LN MTV **(2C)**, and LN TLG **(2D)**. ROC, receiver operator characteristic; SUV, standardized uptake value; MTV, metabolic tumor volume; TLG, total lesion glycolysis; LN, lymph node.

ROC curve analysis was carried out to determine the best cut-off values of SUVmean, SUVmax, MTV, and TLG of the primary lesion of the cervix or positive lymph node in predicting the prognosis of cervical cancer, considering the sensitivity and specificity for survival outcomes (**Figure 2**). The areas under the curves of cervical SUVmean, cervical SUVmax, cervical TLG, and cervical MTV were 0.54 (p=0.544; 95% CI 0.42–0.66), 0.53 (p=0.595; 95% CI 0.41–0.65), 0.57 (p=0.223; 95% CI 0.46–0.69), and 0.57 (p=0.267; 95% CI 0.45–0.68), respectively. The optimal cut-off points of cervical SUVmean, cervical SUVmax, cervical TLG and cervical MTV were 7.9, 12.8, 113.4 and 18.3 cm^3^, respectively. The areas under the curves of LN SUVmean, LN SUVmax, LN TLG, and LN MTV were 0.82 (p=0.002; 95% CI 0.69–0.95), 0.84 (p=0.001; 95% CI 0.71–0.96), 0.77 (p=0.007; 95% CI 0.60–0.95), and 0.72 (p=0.032; 95% CI 0.52–0.92), respectively. The optimal cut-off points of LN SUVmean, LN SUVmax, LN TLG and LN MTV were 2.2, 6.7, 6.8 and 9.8 cm^3^, respectively.

The c-index values of SUVmean, SUVmax, MTV, and TLG of the primary lesion of the cervix or lymph node for OS, DFS, LC and DMFS were shown in **Table 2**. For 125 included patients with cervical cancer, cervical MTV (c-index 0.59-0.61) and cervical TLG (c-index 0.60-0.62) values calculated with a threshold of 40% SUVmax presented stronger prediction capability than cervical SUVmean (c-index 0.51-0.58) and cervical SUVmax (c-index 0.53-0.57) for OS, DFS, LC and DMFS. For 55 cervical cancer patients with positive LN, LN SUVmax presented strongest prediction capability for OS (c-index = 0.79), DFS (c-index = 0.72), LC (c-index = 0.62), and DMFS (c-index = 0.79).

**Table 2:** The c-index values of SUVmean, SUVmax, MTV, and TLG of the primary lesion of the cervix or lymph node for OS, DFS, LC and DMFS.

	C-index			
	OS	DFS	LC	DMFS
**Cervical**				
SUVmax	0.53	0.56	0.57	0.53
SUVmean	0.51	0.56	0.58	0.53
MTV	0.61	0.59	0.59	0.60
TLG	0.61	0.61	0.62	0.62
**Lymph node**				
SUVmax	0.79	0.72	0.62	0.79
SUVmean	0.76	0.63	0.56	0.70
MTV	0.72	0.68	0.58	0.78
TLG	0.77	0.65	0.57	0.71

SUV, standardized uptake value; MTV, metabolic tumor volume; TLG, total lesion glycolysis; OS, overall survival; DFS, disease-free survival; LC, Local control; DMFS, distant metastasis-free survival.

The univariate analysis showed that para-aortic MLNs, total point A EQD_2Gy_, and cervical TLG were significantly associated with DFS (**Table 3**). In multivariate analysis, para-aortic MLNs, total point A EQD_2Gy_ < 85 Gy, and cervical TLG ≥ 113.4 remained significant in predicting DFS (**Table 4**). Furthermore, in multivariate analysis, 2009 FIGO stage, para-aortic MLNs, total point A EQD_2Gy_, and cervical TLG were significant prognostic factors for OS. Para-aortic MLNs was a poor prognostic factor for LC. Para-aortic MLNs, total point A EQD_2Gy_, and cervical TLG had important impacts on DMFS in multivariate analysis. cervical MTV was an important prognostic factor for OS and DMFS in univariate analysis; however, no significant differences were identified for OS and DMFS in multivariate analysis. Moreover, in either univariate or multivariate analyses, cervical SUVmean and cervical SUVmax had no statistically significant correlation with OS, LC, DFS and DMFS.

**Table 3:** Results of the univariate analysis of clinical factors for disease-free survival.

Variable	Univariate analysis		
	HR	95%CI	P value
**Age (continuous, year)**	1.003	0.969-1.038	0.882
**2009 FIGO stage**			
I-II *vs.* III-IV	2.114	0.988-4.523	0.054
**Histology**			
Squamous *vs.* non-squamous	2.459	0.939-6.440	0.067
**Primary tumor size**			
≤4 cm *vs.* >4 cm	1.336	0.651-2.738	0.429
**Pelvic MLNs**			
Negative *vs.* Positive	1.368	0.667-2.805	0.393
**Para-aortic MLNs**			
Negative *vs.* Positive	6.166	2.711-14.024	< 0.001
**Treatment duration**			
≤56 days *vs.* >56 days	1.785	0.835-3.818	0.135
**Total point A EQD_2Gy_**			
<85 Gy *vs.* ≥85 Gy	0.382	0.155-0.942	0.037
**Cycles of concurrent chemoradiotherapy**			
<4 *vs.* ≥4	0.839	0.360-1.956	0.684
**Cervical SUVmean**			
<7.9 *vs.* ≥7.9	1.555	0.759-3.187	0.227
**Cervical SUVmax**			
<12.8 *vs.* ≥12.8	1.788	0.867-3.688	0.116
**Cervical MTV**			
<18.3 cm^3^ *vs.* ≥18.3 cm^3^	2.095	0.980-4.480	0.056
**Cervical TLG**			
<113.4 *vs.* ≥113.4	2.629	1.169-5.914	0.019

HR, hazard ratio; CI, confidence interval; FIGO, International Federation of Gynecology and Obstetrics; MLN, metastatic lymph node; EQD_2Gy_, equivalent dose at 2 Gy; SUV, standardized uptake value; MTV, metabolic tumor volume; TLG, total lesion glycolysis.

**Table 4:** Results of the multivariate analysis of clinical factors for disease-free survival.

Variable	Multivariate analysis	
	HR (95% CI)	P value
**Para-aortic MLNs**		
Yes	Reference	
No	0.116 (0.048-0.278)	<0.001
**Total point A EQD_2Gy_**		
≥85 Gy	Reference	
<85 Gy	3.296 (1.316-8.253)	0.011
**Cervical TLG**		
≥113.4	Reference	
<113.4	0.278 (0.121-0.640)	0.003

HR, hazard ratio; CI, confidence interval; MLN, metastatic lymph node; EQD_2Gy_, equivalent dose at 2 Gy; TLG, total lesion glycolysis.

**Figure 3:**
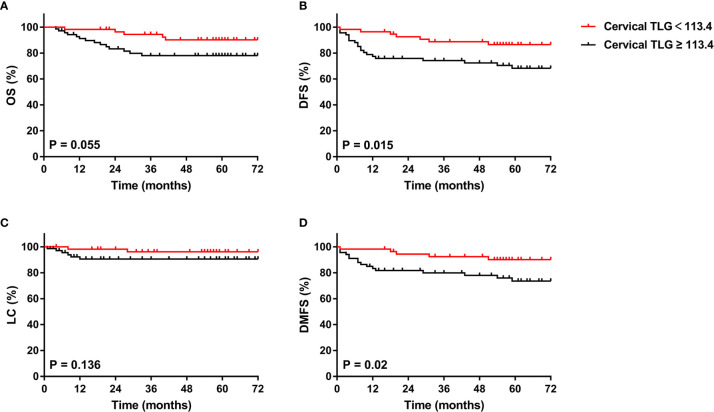
Kaplan-Meier curves of OS **(A)**, DFS **(B)**, LC **(C)**, and DMFS **(D)** of all included patients with cervical cancer for cervical TLG. OS, overall survival; DFS, disease-free survival; LC, local control; DMFS, distant metastasis-free survival.

The 5-year overall survival, disease-free survival, local control and distant metastasis-free survival rates for patients with cervical TLG levels <113.4 and ≥ 113.4 were 90.1% and 78% (p=0.055), 86.5% and 66.1% (p=0.015), 96.2% and 89.3% (p=0.136), and 90.9% and 71.2% (p=0.02), respectively (**Figure 3**). For the 70 patients with cervical TLG ≥ 113.4, the median follow-up period for all patients was 61 months (range, 4-110 months). The median DFS period was 56 months. Of these patients, 22 patients (31.4%) suffered from disease failure, including 4 patients with pelvic recurrence, 15 patients with distant metastases, 3 patients with concurrent local and distant progression. The total local recurrence and distant metastases rates were 10% and 25.7%, respectively. Of the 22 patients who suffered from disease failure, disease progression occurred within 2 years after primary treatment in 18 patients (81.8%) and within 5 years after primary treatment in all patients (100%). The 5-year OS, DFS, LC and DMFS rates for patients with cervical MTV levels <18.3 cm^3^ and ≥ 18.3 cm^3^ were 90.9% and 76.6% (p=0.03), 83.5% and 67.7% (p=0.051), 94.7% and 90.3% (p=0.32), and 88.4% and 71.8% (p=0.031), respectively.

**Figure 4:**
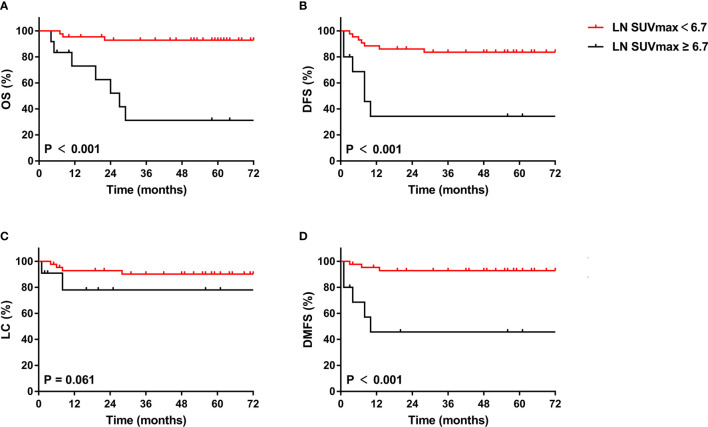
Kaplan-Meier curves of OS **(A)**, DFS **(B)**, LC **(C)**, and DMFS **(D)** of cervical cancer patients with positive LN for LN SUVmax. OS, overall survival; DFS, disease-free survival; LC, local control; DMFS, distant metastasis-free survival.

Of the 125 included patients, 55 had pelvic or para-aortic nodal involvement. The 5-year OS, DFS, LC and DMFS rates for patients with positive LN were 80.4%, 72.2%, 86.1% and 81.4%, respectively. In univariate analysis, LN SUVmean, LN SUVmax, LN MTV, LN TLG, 2009 FIGO stage, para-aortic MLNs, treatment duration, and cycles of concurrent chemoradiotherapy were significantly associated with OS; LN SUVmax, LN MTV, LN TLG, 2009 FIGO stage, para-aortic MLNs, and treatment duration were significantly associated with DFS; LN SUVmean, LN SUVmax, LN MTV, LN TLG, 2009 FIGO stage, and para-aortic MLNs were significantly associated with DMFS; and only para-aortic MLNs was significantly associated with LC. In multivariate analysis, LN SUVmax remained significant biomarker linked to OS, DFS, and DMFS, and LN MTV was also connected with DMFS. The Kaplan-Meier curves of OS, DFS, LC, and DMFS for LN SUVmax were shown in **Figure 4**. However, SUVmean, SUVmax, MTV, and TLG of the primary lesion of the cervix had no correlation with survival outcomes in patients with positive LN.

## Discussion

At present, various metabolic parameters of PET, such as MTV and TLG, have particularly become a research hotspot for predicting the prognosis of cervical cancer. However, there are different opinions on the role of PET metabolic parameters in the prognosis of cervical cancer. Some studies have shown that metabolic parameters of PET, such as SUVmax, SUVmean, MTV and TLG, play an important role in predicting the prognosis of cervical cancer (**Table 5**). However, other studies have found no significant correlation between these parameters and survival. Therefore, in our study, we investigated the relationships between clinical characteristics and PET metabolic parameters and the recurrence and long-term survival of cervical cancer. Our study shows that pre-treatment cervical TLG, LN SUVmax, Para-aortic MLNs, and Total point A EQD_2Gy_ are important independent prognostic factors for recurrence and survival.

**Table 5:** Previous studies regarding pre-treatment metabolic parameters in patients with locally advanced cervical cancer.

Authors (year)	Design	N	FIGO stage	LN+ patients	Treatment	Statistical analysis	Pre-treatment Cervical metabolic parameters associated with survival outcomes	Pre-treatment LN metabolic parameters associated with survival outcomes
Voglimacci et al. (15)	R	93	IB2-IVA	33	CRT	Univariate analysis	SUVmax: OS and RFS	–
						Multivariate analysis	SUVmax: OS	–
Herrera et al. (19)	R	38	IB1-IVA	22	CRT	Univariate analysis	SUVmean: OS, DFS, and LRC TGV: OS, DFS, and LRC	–
						Multivariate analysis	TGV: OS and DFS	–
Leseur et al. (13)	P	53	IB2-IVA	Unknown	CRT/RT	Univariate analysis	MTV: OS and DFS TLG: OS and DFS	–
						Multivariate analysis	MTV: OS and DFS	–
Hong et al. (22)	R	56	IIB-IVA	51	CRT	Univariate analysis	MTV: RFS TLG: RFS	–
						Multivariate analysis	TLG: RFS	–
Martinez et al. (21)	R	125	IB2-IVA	47	CRT	Univariate analysis	MTV: para-aortic LN involvement TLG: para-aortic LN involvement	SUVmean: para-aortic LN involvement SUVmax: para-aortic LN involvement Pelvic LN/Cervical Tumor SUVmax ratio: para-aortic LN involvement MTV: para-aortic LN involvement TLG: para-aortic LN involvement
						Multivariate analysis	MTV: para-aortic LN involvement	–
Sun et al. (26)	R	91	IB1-IVB	26	Sur/Sur+CRT or ChT/CRT	Univariate analysis	SUVmax: OS MTV: OS TLG: OS	–
						Multivariate analysis	MTV: OS	–
Guler et al. (27)	R	129	IB2-IVA	76	CRT	Univariate analysis	SUVmean: OS and DFS SUVmax: OS and DFS MTV: OS and DFS TLG: OS and DFS	–
						Multivariate analysis	–	–
Yoo et al. (28)	R	73	I-IVB	28	Sur/sur+CRT/CRT/RT/ChT	Univariate analysis	MTV: DFS TLG: DFS	–
						Multivariate analysis	TLG: DFS	–
Liang et al. (29)	R	67	IB-IVA	Unknown	Sur/Sur+CRT or RT/ CRT/ChT	Univariate analysis	Total SUVmax: OS and PFS Total MTV: OS and PFS Total TLG: OS and PFS Total TLG: OS and PFS	
						Multivariate analysis	
Carpenter et al. (18)	P	30	IB1-IVA	24	CRT	Univariate analysis	Total MTV: OS, DFS, and DM Total TLG: OS and DM	
						Multivariate analysis	–	
Lima et al. (17)	R	82	IIA-IVA	44	CRT	Univariate analysis	Total MTV: response to therapy Total TLG: response to therapy	
						Multivariate analysis	Total MTV: response to therapy	
Our study	R	125	IB-IVA	55	CRT/RT/NAC + CRT or RT	Univariate analysis	TLG: DFS and DMFS	SUVmean: OS SUVmax: OS, DFS, and DMFS MTV: OS, DFS, and DMFS TLG: OS, DFS, and DMFS
						Multivariate analysis	TLG: OS, DFS, and DMFS	SUVmax: OS, DFS, and DMFS MTV: DMFS

N, number of patients; R, retrospective; P, prospective; FIGO, International Federation of Gynecology and Obstetrics; LN, lymph node; NAC, neoadjuvant chemotherapy; CRT, chemoradiotherapy; RT, radiotherapy; ChT, chemotherapy; Sur, surgery; SUV, standardized uptake value; MTV, metabolic tumor volume; TLG, total lesion glycolysis; TGV, tumor glycolytic volume; age, average; SD, standard deviation; OS, overall survival; RFS, relapse-free survival; DFS, disease-free survival; PFS, progression-free survival; LRC, Loco-regional control; DMFS, distant metastasis-free survival; DM, distant metastasis.

SUV can reflect metabolic activity as a semiquantitative marker of tumor uptake and has been demonstrated to play an important role in predicting the prognosis of cervical cancer in previous studies. A meta-analysis demonstrated that a significantly worse prognosis was associated with a higher SUVmax of the primary lesion in cervical cancer. However, SUVmax was not a significant independent prognostic factor in most of the enrolled studies in that meta-analysis (14). There are various reasons for this contradictory result, especially publication bias, which cannot be ignored. In addition, there are several limitations, such as missing data, the small sample size of each enrolled study and inconsistent treatment methods in different medical centers, which may cause differences in results. Voglimacci et al. (15) also suggested that cervical SUVmax as a continuous variable was a critical predictive index for survival outcomes, but the difference was not statistically significant when using the cut-off value. Herrera et al. (19) reported that pre-treatment cervical SUVmean ≥ 5 was a significantly poor prognostic factor of OS (57% *vs*. 86%, p =0.03), DFS (36% *vs*. 88%, p = 0.004) and LC (65% *vs*. 88%, p = 0.04) in univariate analysis. However, statistically significant associations were not found between cervical SUVmean and survival outcomes in multivariate analysis. Meanwhile, the demonstration of an association between cervical SUVmax and prognosis would have been more challenging to interpret. In our study, cervical SUVmean and cervical SUVmax had no statistically significant correlation with OS, DFS, LC or DMFS. There are several articles with similar results to our study (13, 22). However, LN SUVmax was significant associated with survival outcomes. Similarly, Martinez et al. (21) indicated that LN SUVmax was significantly linked to para-aortic nodal involvement only in univariate analysis. SUV may be affected by many factors, including blood glucose level, body mass index, scan duration, and reconstruction algorithm (23–25). Therefore, the role of SUVmean and SUVmax in predicting the prognosis of cervical cancer is still controversial and remains to be further studied.

MTV represents the volume of metabolically active malignant lesions, which is similar but more accurate than the measurement of tumor size on physical examination and may be significantly correlated to the prognosis of the disease. Leseur et al. (13) demonstrated that cervical MTV calculated with a segmentation of 55% SUVmax from pre-treatment PET/CT was applicable for predicting patient survival outcomes after concurrent chemoradiotherapy for patients with locally advanced cervical cancer. Similarly, Sun et al. (26) also considered that cervical MTV accumulation with a threshold of 40% SUVmax was a critical prognostic factor for cervical cancer patients and should be used to guide oncologists in selecting individualized therapies. Martinez et al. (21) proposed that cervical MTV calculated with a threshold of 40% SUVmax was an independent prognostic biomarker on para-aortic nodal involvement prediction. Guler et al. (27) took the opposite view that the role of using cervical MTV, calculated with the primary cervical tumor equal to or greater than an SUV of 2.5, to predict the prognosis of patients with cervical cancer and to develop patient treatment strategies required further confirmation. In our study, cervical MTV, calculated with a threshold of 40% SUVmax, presented an obvious association with OS but failed to reach the 0.05 significance level for DFS in univariate analysis; however, there was no significant association between cervical MTV and OS in multivariate analysis. For cervical cancer patients with positive LN, LN MTV remained significant correlation with DMFS in multivariate. We considered that the reasons for these different results may be related to the inconsistency in the definition of MTV in different studies. Therefore, we believe that MTV alone is not rigorous enough to predict the prognosis of patients with cervical cancer in the absence of a consistent definition of MTV.

The combination of MTV and TLG is a more resultful prognostic factor that takes into consideration both tumor volume and metabolic activity as crucial parameters of tumor response to treatment. Yoo et al. (28) highlighted that cervical TLG (cut-off, 7600), a volume-based metabolic parameter for primary cervical tumors, was a significant predictor of recurrence in cervical cancer in both univariate analysis and multivariate analysis. Likewise, Liang et al. (29) reported that total TLG was obviously correlated with survival outcomes in patients with locally advanced cervical cancer. Similarly, Carpenter et al. (18) indicated that total TLG measured by ^18^F-FDG PET/CT was correlated with OS in high-risk cervical cancer patients treated with chemoradiotherapy and brachytherapy. Lima et al. (17) also preliminary suggested that although its p value seems to be below the critical value, pre-treatment total TLG was a significant independent predictor of response to therapy. However, the sample sizes of these studies were less than 100 cases. In our group, we obtained similar results and had a larger sample size. Although TLG confronts the same challenges as MTV, we still believe that the combination of multiple parameters makes predictions more effective.

In our study, we found that distant metastasis in patients with LACC treated with chemoradiotherapy and brachytherapy was a major pattern of treatment failure. This finding was consistent with that of previous research. Importantly, we found that cervical TLG and LN SUVmax were important prognostic factors for OS, DFS, and DMFS. The role of additional chemotherapy included adjuvant chemotherapy or neoadjuvant chemotherapy is still controversial. Dueñas-González et al. (30) investigated 515 patients with locally controlled cervical cancer in a randomized study. The results showed that the 3-year PFS of concurrent chemoradiotherapy following two adjuvant cycles of cisplatin plus gemcitabine was significantly improved compared with standard therapy (74.4% *vs* 65.0%, p=0.029); the same result was found for OS (log-rank p= 0.0224; HR, 0.68; 95% CI, 0.49 to 0.95). However, the intervention group had more grade 3 and 4 toxicities than the control group (p<0.001). Adjuvant chemotherapy has not been widely accepted because further studies are needed to demonstrate the contributions of multiagent chemoradiotherapy and adjuvant chemotherapy to survival outcomes, and toxicity cannot be ignored. Da Costa et al. (31) conducted a randomized phase II trial to evaluate the efficacy and safety of neoadjuvant chemotherapy (NAC) followed by concurrent chemoradiotherapy (CRT) and reported that the addition of NAC with cisplatin and gemcitabine to CRT is not superior to standard CRT alone for LACC. Additionally, a phase III multicenter trial of weekly induction chemotherapy consisting of paclitaxel and carboplatin followed by standard CRT versus standard CRT alone in patients with LACC is undergoing (NCT01566240).

Local recurrence in cervical cancer patients treated with concurrent chemoradiotherapy or radiotherapy was another pattern of treatment failure. MRI-guided adaptive brachytherapy, which plays an important role, increased the radiation dose to the tumor and led to a significant improvement in the local control rate while minimizing the radiation dose delivered to surrounding normal tissues (32, 33). In our study, we also found that for the 22 patients with cervical TLG ≥ 113.4 who experienced treatment failure, disease recurrence occurred in all patients within 5 years after treatment. Thus, active follow-up for at least 5 years is essential. These findings may provide an early signal-individualized intensive therapeutic approach with either adjuvant chemotherapy or MRI-guided adaptive brachytherapy.

The present study demonstrates the value of the metabolic parameters of pre-treatment ^18^F-FDG PET/CT as prognostic factors in patients with LACC. However, this study has several limitations. Most notably, this is a retrospective study with a small number of included patients and baseline data are easily to be incomplete. Moreover, this study includes a long-time span and changes in the treatment strategies may affect the results. In addition, positive LNs are identified on PET/CT and not by histopathologic verification. We cannot confirm that all FDG-avid LNs are histopathological lymphadenopathies. Finally, recently there are many promising methods such as radiomics, machine learning, and especially deep learning (34, 35). Combining these promising image analysis techniques may have more significant predictive values for the prognosis of cervical cancer. Further prospective randomized clinical trials with a large number of patients are required to evaluate the value of the metabolic parameters in survival outcomes prediction.

## Conclusion

Pre-treatment cervical and lymph node metabolic parameters were associated with survival outcomes in patients with LACC. In our study, we found that pre-treatment cervical TLG and lymph node SUVmax may be important prognostic biomarkers for OS, DFS, and DMFS in patients with LACC. However, further prospective studies with a large number of patients are required to evaluate the value of the metabolic parameters in survival outcomes prediction.

## Disclosure

The abstract of this paper was presented at the 2020 ASTRO Conference name “The Role of Metabolic Parameters of Pre-treatment ^18^F-FDG PET/CT in Patients with Locally Advanced Cervical Cancer” as a poster presentation with interim findings. The poster’s abstract was published in “Poster Q&A Session” in International Journal of Radiation Oncology*Biology*Physics Journal name “The Role of Metabolic Parameters of Pre-treatment ^18^F-FDG PET/CT in Patients with Locally Advanced Cervical Cancer”: Hyperlink with DOI (https://doi.org/10.1016/j.ijrobp.2020.07.1572).

## Data Availability Statement

Data used in this study are not publicly available and can only be accessed, with appropriate approvals from data custodians and ethical clearance, from Peking Union Medical College Hospital. Requests to access the datasets should be directed to FZ, zhangfq@pumch.cn.

## Ethics Statement

The studies involving human participants were reviewed and approved by Peking Union Medical College Hospital Ethics Committee [Protocol number S-K 1645]. The patients/participants provided their written informed consent to participate in this study.

## Author Contributions

FZ and KH contributed to the conception and the design of the study. DW wrote the first draft of the manuscript. WW and XL contributed to the review of literatures. LH and QP organized the database. XR performed the statistical analysis. All authors contributed to the article and approved the submitted version.

## Funding

This study was funded by the National Key Research and Development Plan, the Ministry of Science and Technology of the People’s Republic of China [grant number 2016YFC0105207] and the National Natural Science Foundation of China [grant number U19A2064] and the CAMS Innovation Fund for Medical Sciences (CIFMS) [grant number 2020-I2M-C&T-B-036].

## Conflict of Interest

The authors declare that the research was conducted in the absence of any commercial or financial relationships that could be construed as a potential conflict of interest.

## Publisher’s Note

All claims expressed in this article are solely those of the authors and do not necessarily represent those of their affiliated organizations, or those of the publisher, the editors and the reviewers. Any product that may be evaluated in this article, or claim that may be made by its manufacturer, is not guaranteed or endorsed by the publisher.
